# Involvement of Increased Endogenous Asymmetric Dimethylarginine in the Hepatic Endoplasmic Reticulum Stress of Type 2 Diabetic Rats

**DOI:** 10.1371/journal.pone.0097125

**Published:** 2014-06-11

**Authors:** Yi-Ping Leng, Ni Qiu, Wei-jin Fang, Mei Zhang, Zhi-Min He, Yan Xiong

**Affiliations:** 1 Department of Pharmacology, Guangzhou Research Institute of Snake Venom and School of Pharmaceutical Sciences, Guangzhou Medical University, Guangzhou, Guangdong, P.R. China; 2 Department of Pharmacology, School of Pharmaceutical Sciences, Central South University, Changsha, Hunan, P.R. China; 3 Cancer Research Institute and Cancer Hospital, Guangzhou Medical University, Guangzhou, Guangdong, P.R. China; University Dresden, Germany

## Abstract

**Objective:**

Increasing evidence suggested that endoplasmic reticulum (ER) stress contributes to insulin resistance, which plays an important role in the development of type 2 diabetes mellitus (T2DM). Accumulation of endogenous nitric oxide synthase (NOS) inhibitor, asymmetric dimethylarginine (ADMA), is associated with insulin resistance, T2DM, and diabetic cardiovascular complications, although the mechanisms have not been elucidated. This study was to determine whether elevated endogenous ADMA is involved in hepatic ER stress of type 2 diabetic rats, verify their causal relationship, and elucidate the potential mechanism underlying ADMA induced ER stress in rat hepatocytes.

**Methods:**

Immunoglobulin binding protein (Bip) transcription, eukaryotic initiation factor 2α kinase (eIF2α) phosphorylation, X box-binding protein-1 (XBP-1) mRNA splicing and C/EBP homologues protein (CHOP) expression were measured to reflect ER stress. Contents of ADMA and nitrite/nitrate as well as activities or expression of NOS and dimethylarginine dimethylaminohydrolase (DDAH) were detected to show the changes in DDAH/ADMA/NOS/NO pathway. The lipid peroxidation product malondialdehyde content and antioxidant enzyme superoxide dismutase activity were analyzed to evaluate oxidative stress.

**Results:**

ER stress was provoked in the liver of type 2 diabetic rats, as expressed by increases of Bip transcription, eIF2α phosphorylation, XBP-1 splicing and CHOP expression, all of which were in parallel with the elevation of serum ADMA, suppression of NO generation, NOS and DDAH activities in the liver. Exposure of hepatocytes to ADMA or hydrogen peroxide also induced ER stress, which was associated with the inhibition of NO production and increase of oxidative stress. Treatment of hepatocytes with antioxidant pyrrolidine dithiocarbamate not only decreased ADMA-induced oxidative stress and inhibition of NO production but also reduced ADMA-triggered ER stress.

**Conclusions:**

These results indicate that increased endogenous ADMA contributes to hepatic ER stress in type 2 diabetic rats, and the mechanism underlying ADMA-induced ER stress may relate to oxidative stress via NOS uncoupling.

## Introduction

Diabetes mellitus is one of the most prevalent and serious metabolic diseases in the world. It is mainly classified into type 1 diabetes and type 2 diabetes, and 90∼95% diabetic patients are type 2 diabetes mellitus, which is characterized by insulin resistance that results from the decrease in insulin action on target tissues. Liver as the major target organs of insulin plays important roles in the development of insulin resistance and type 2 diabetes mellitus, and the underlying mechanisms are still not fully understood. Increasing evidence demonstrate that endoplasmic reticulum (ER) stress has emerged as an important player in the onset of hepatic insulin resistance [1] and diabetes mellitus [2].

As a eukaryotic organelle, ER plays important roles in protein synthesis, folding and transport, calcium homoeostasis, and lipid synthesis. The change of calcium homeostasis and cellular redox state may result in the failure of protein folding in ER, these conditions that disturb ER functions are called ER stress. The accumulation of unfolded protein or misfolded protein in ER stimulates the upregulation of ER chaperones 78 kDa glucose-regulated protein (GRP78), also termed as the binding immunoglobulin protein (Bip), which may increase the protein folding activity and prevent the protein aggregation [3]. On the other hand, the activation of the protein kinase R-like ER kinase (PERK) results in the phosphorylation of eukaryotic initiation factor 2α kinase (eIF2α), which can shut off global mRNA translation and reduce the protein-folding load on the ER [4]. When the ER could not dispose the increased amount of unfolded protein, the activation of type I transmembrane protein kinase/endoribonuclease (IRE1) and X box-binding protein-1 (XBP-1) mRNA splicing would occur, resulting in the expression of ER degradation enhancer mannosidase alpha-like protein and facilitation of the ER-related degradation [5]. Once the above process could not maintain the balance of protein synthesis and folding, the apoptosis of some cells would be generated to ensure the normal function of other cells. It has been well known that the excessive expression of C/EBP homologus protein (CHOP) plays an important role in ER stress-induced apoptosis [6]. Therefore, the transcription of Bip, phosphorylation of eIF2α, splicing of XBP-1 mRNA and expression of CHOP could be used as the markers of ER stress.

Recent investigations have demonstrated that ER stress facilitates insulin resistance and β cell apoptosis [2], [7]. Elevated IRE-1 phosphorylation can lead to an increase of tumor necrosis factor receptor–associated factor 2 expression and activation of the c-jun amino-terminus kinase and serine kinase [8], which promote the serine phosphorylation of insulin receptor substrate-1, inhibited phosphatidylinositol-3 mediated insulin signal transduction and reduced the sensitivity of target tissues to insulin [9]. To reduce ER stress by the treatment of sodium phenylbutyrate, a chemical chaperone could ameliorate lipid-induced insulin resistance and β-cell dysfunction in obese nondiabetic humans [10]. Oxidative stress and calcium depletion are considered as the major triggers of ER stress. Excessive reactive oxygen species (ROS) generated under oxidative stress may oxidize sarcoplasmic/ER Ca^2+^-ATPase on ER membrane, resulting in the decreases of calcium concentration in ER, and thus affecting the activity of calcium-dependent protein folding enzyme [11]. Furthermore, the maintenance of some protein folding enzymes activity needs free sulfhydryl, which will be attacked by superoxide, resulting in the inhibition of protein folding and aggravating ER stress [12]. Therefore, treatment with antioxidant or free radical scavenger could inhibit ER stress-induced apoptosis [13]–[15].

Accumulating studies have demonstrated that endogenous asymmetric dimethylarginine (ADMA) plays important roles in insulin resistance [16], [17], diabetes [18], [19] and diabetic cardiovascular complication [20]. Endogenous ADMA was derived from proteins containing methylated arginine residues via hydrolysis, and its major metabolic pathway is degraded by the enzyme dimethylarginine dimethylaminohydrolase (DDAH) to dimethylamine and L-citrulline. It is well documented that ADMA not only inhibits NOS activity, decreasing NO synthesis, but also causes NOS uncoupling, increasing superoxide production, resulting in the enhance of oxidative stress [21], which is closely related to ER stress. Therefore, it is very important to determine the relationship between endogenous ADMA and ER stress in diabetes mellitus. The present study was designed to investigate whether elevated endogenous ADMA is involved in hepatic ER stress in type 2 diabetic rats and further to verify their causal relationship and elucidate the potential mechanism underlying ADMA induced ER stress in cultured rat hepatocytes. This study will provide some new insight into the pathogenesis of type 2 diabetes mellitus and comprehensive pathogenic roles of ADMA.

## Materials and Methods

### 1. Reagents

N^G^-Nitro-L-arginine methyl ester (L-NAME), ADMA and pyrrolidine dithiocarbamate (PDTC) were purchased from Sigma Chemical Co. (St. Louis, MO, USA). Thiobarbituric acid was from Fluka (Milwaukee, WI, USA). Dulbecco’s modified Eagle’s medium (DMEM), fetal bovine serum (FBS) and TRIzol were purchased from Gibco (Gaithersburg, MD, USA). The polyclonal antibody against DDAH2 or DDAH1 was purchased from Abcam (Cambridge, MA, USA) while the polyclonal antibody against total eIF-2α or phosphor- eIF-2α, CHOP and β-actin were obtained from Cell Signaling Technology (Boston, MA, USA). The kits for measurement of nitrite/nitrate, malondialdehyde (MDA), protein content and NOS, superoxide dismutase (SOD) activity were products of Nanjing Jiancheng Bioengineer Institute (Jiangsu, China), while the commercial kits for assays of total cholesterol (TC), triglyceride (TG), high density lipoprotein-cholesterol (HDL-C) and low density lipoprotein-cholesterol (LDL-C) level were products of Zhongsheng Bioengineering Company (Beijing, China). The insulin radioimmunoassay kit was purchased from Atom Hightech Co., Ltd (Beijing, China).

### 2. Preparation of Type 2 Diabetic Rat Model

The animal experiments were approved by the Animal Care and Use Committee of Central South University and Guangzhou Medical University. Sprague-Dawley rats from the Animal Center of Central South University were assigned to normal chow (Control rats) or high-fat diet (60% chow, 10% Egg yolk powder, 10% lard, 1.5% cholesterol and 0.1% sodium cholate). After 4 weeks high-fat diet feeding, the rats were injected intraperitoneally of a low dose streptozotocin (35 mg/kg, i.p) to induce a moderate pancreatic injury and continued high-fat feeding for 8 weeks (Type 2 diabetic rats) as previously described [19]. All animals were caged with free access to food and water in an air-conditioned room under a 12 h light (7 AM to 7 PM)/12 h dark (7 PM to 7 AM) cycle. At the end of the experiment, rats were anaesthetized with sodium pentobarbital (30 mg/kg, i.p.), and blood were collected via carotid artery intubation for separating serum or plasma. After the animal was died from the blood loss, the liver was then quickly excised to freeze in liquid nitrogen and subsequently store at −70°C for the determination of gene expression and biochemical assays.

### 3. Metabolic Assays

An oral glucose tolerance test (OGTT) was performed 3 days before the end of experiment to evaluate insulin sensitivity. At the beginning of OGTT, the levels of fasting blood glucose (FBG) were measured using a glucometer (One Touch, Johnson and Johnson, Milpitas, CA) and blood sample taken from the tail vein of rat (at 0 min) after fasting 12 h. Subsequently, rats were given a 40% glucose solution (2.0 g/kg) with gavage and then four additional blood samples were collected at 30, 60, 90 and 120 min after glucose gavage for measurements of glucose concentrations. The area under the curve (AUC) of blood glucose concentration were calculated using proximity ladder shaped formula [22]. The concentration of fasting plasma insulin (FIns) was determined by a radioimmunoassay technique. Insulin sensitivity index (ISI) was calculated with FBG and FIns (ISI = 1/FBG×FIns). Serum lipid profiles including TC, TG, HDL-C and LDL-C were assayed spectrophotometrically using the respective commercial kits.

### 4. Cell Culture and Experiment Design

Rat hepatoma cells (H4IIE, ATCC, Manassas, USA) were cultured in Dulbecco’s modified Eagle’s medium which was supplemented with 10%FBS at 37°C and a humidified atmosphere of 5% CO_2_. In experiments of dose-response and time course, the cells were incubated with various concentration ADMA (0, 10, 30, 100 µmol/L) for different time (0, 24, 48, 72 h). According to the experiment results, the treatment dose and acting duration of ADMA in subsequent experiments were determined. The cells were divided into eight groups and treated with hydrogen peroxide (0.5 mmol/L, as positive control of ER stress), ADMA (30 µmol/L), another exogenous NOS inhibitor L-NAME (30 µmol/L), antioxidant PDTC (10 µmol/L) alone, ADMA plus PDTC, L-NAME plus PDTC (10 µmol/L) and without any drug (as normal control) for 48 h, respectively. But the treatment with hydrogen peroxide only for 1 h, and in the groups of combined treatment, PDTC was added for 2 h before ADMA or L-NAME treatment. Cells from all groups were harvested to store in −70°C for RT-PCR assays and activities of NOS and SOD. The media were collected to store in −70°C for the measurements of MDA and nitrite/nitrate contents.

### 5. RT-PCR Analysis and Evaluation of XBP-1 Processing

Total RNA was extracted from liver and cultured cells with Trizol reagent (Invitrogen, Carlsbad, CA, USA) and reversely transcribed into cDNA using AMV reverse transcriptase kit (Promega, Madison, USA). The resulting cDNA samples were PCR amplified with specific primers (Table 1) of rat Bip, XBP-1, CHOP, DDAH1 and DDAH2 genes at the respective reaction condition (Table 2). The amplified fragments were then separated on 1.5% agarose gels and visualized by ethidium bromide staining. The optical densities of mRNA bands were quantified with Gel-pro analyzer (Media Cybernetics, Inc. Bethesda, MD, USA) and normalized to GAPDH or β-actin as an internal control.

**Table 1:** Primers used in the study.

Genes	Primer sequence		PCR Products
Bip	Sense	5′-AGCCCACCGTAACAATCAAG-3′	445 bp
Bip	Antisense	5′-TCCAGCCA TTCGATCTTTTC-3′	
XBP-1	Sense	5′-AAACAGAGTAGCAGCACAGACTG-3′	601 bp
XBP-1	Antisense	5′-GATCTCTAAGACTAGAGGCTTGGTG-3′	
CHOP	Sense	5′-GAGTCTCTGCCTTTCGCCTT-3′	373 bp
CHOP	Antisense	5′-TCTCATTCTCCTGCTCCTTCTC-3′	
DDAH1	Sense	5′-AGGACAAATCAACGAGGTGC-3′	305 bp
DDAH1	Antisense	5′- TTTGC GCTTTCTGGGTACTC-3′	
DDAH2	Sense	5′-GGAGGTAAACTGAGGC AACG-3′	537 bp
DDAH2	Antisense	5′-GGAGGTAAACTGAGGC AACG-3′	
β-actin	Sense	5′-TCTTCTGGGAGGTAGCAGGA-3′	241 bp
β-actin	Antisense	5′-CACGATGGAGGGGCCGGACTCATC-3′	
GAPDH	Sense	5′- AACTTTGGCATTGTGGAAGG-3′	662 bp
GAPDH	Antisense	5′- TGTGAGGGAGATGCTCAGTG-3′	

**Table 2:** Reaction condition of polymerase chain reaction.

Genes	Initial denature	Cycle parameters	Final extension	Cycle number
Bip	94°C 5 min	94°C 30 s, 53°C 30 s, 72°C 30 s;	72°C 10 min	30
XBP-1	94°C 5 min	90°C 60 s, 60°C 60s, 70°C 60 s,	72°C 10 min	30
CHOP	94°C 5 min	94°C 30 s, 52.2°C 30 s, 72°C 30 s,	72°C 10 min	30
DDAH1	94°C 5 min	94°C 45 s, 55°C 45 s, 72°C 45 s,	72°C 10 min	30
DDAH2	94°C 5 min	94°C 45 s, 64°C 45 s, 72°C 45 s,	72°C 10 min	30

It has been well established that IRE-1α activation induced the cleavage of XBP-1 mRNA [23]. XBP-1 processing is characterized by excision of a 26-bp sequence from the coding region of XBP-1 mRNA. The cleaved fragment contains a Pst I restriction site, and the extent of XBP-1 processing thus can be evaluated by restriction analysis. A fragment of 601 bp of XBP-1 cDNA, encompassing the 26-bp excised region, was amplified by conventional PCR and purified and then incubated with Pst I restriction enzyme for 5 h at 37°C. PCR products derived from nonspliced XBP-1 mRNA (indicating absence of ER stress) were digested in two approximative bands of 300 bp. In contrast, products amplified from spliced XBP-1 mRNA were resistant to digestion and remained 601 bp long, indicating presence of ER stress.

### 6. Western Blotting for Protein Expression

Liver tissues lysates were prepared by homogenization with RIPA buffer as described previously [24], and equivalent amounts of protein(10–20 µg/well) were loaded on 10% sodium dodecyl sulfate polyacrylamide gel electrophoresis and the proteins were transferred onto a PDVF membrane. The membranes were incubated with 5% skimmed milk at room temperature for 1 h, and then incubated with polyclonal antibody against DDAH1 or DDAH2, total eIF-2α or phosphor- eIF-2α, CHOP and β-actin over night at 4°C, followed by incubation with second goat anti-rabbit polyclonal antibody at room temperature for 1h, respectively. After washing again, protein bands were detected with the method of chemiluminescence and visualized with a Molecular Imager^®^ ChemiDoc™ XRS+ System (Bio-Rad Laboratories, Hercules, CA, USA).

### 7. Assay of DDAH, NOS, and SOD Activities

Livers from each group were homogenized in ice-cold phosphate buffer solution (0.1 mol/L, pH 6.5) and centrifuged at 3000 g for 15 min (4°C). The supernatant was used for assay of DDAH, NOS, and SOD activity. Cells from each group were lysed in 300 µl ice-cold lysis buffer (50 mM Tris-HCl PH 7.4, 150 mM NaCl, 1 mM EDTA, 1% Triton X-100, 1% sodium deoxycholate, 0.1% SDS, temporarily supplemented with 1 mM PMSF) for 30 min and then harvested for centrifuging at 10,000 g for 60 min (4°C) to remove the nuclei and large debris. The supernatant was used for assay of NOS and SOD activity with the respective commercial kit.

The activity of DDAH was assayed by determining L-citrulline formation in tissue homogenates as previously described [25]. One unit of the enzyme activity was defined as the amount that catalyzed formation of 1 µmol/L L-citrulline from ADMA per min at 37°C. NOS activity was assayed by the conversion of L-arginine to NO with a commercial kit in tissue homogenates or cell lysates as previously described [26]. One unit of the enzyme activity was defined as the amount that catalyzed formation of 1 nmol NO from L-arginine per min at 37°C. SOD activity was determined by monitoring the inhibition of the autoxidation of hydroxylamine in tissue homogenates or cell lysates as previously described [27]. One unit of the enzyme activity was defined as the amount that inhibited autoxidation of hydroxylamine by 50%.

### 8. Measurements of ADMA, Nitrite/nitrate and MDA Levels

Serum ADMA concentration was measured by high-performance liquid chromatography as previously described [27]. Serum (0.1 ml) mixed with 5-sulpfosalicylic acid (2 mg), and the mixture was stored at 4°C for 10 min. The precipitated protein was removed by centrifugation at 2500 g for 15 min (4°C), and the supernatant was used for the measurement of ADMA. The level of NO in tissue homogenates or cell conditioned media was reflected indirectly by the content of nitrite/nitrate, the stable end product of NO. The contents of nitrite/nitrate were determined by converting nitrate into nitrite with aspergillus nitrite reductase [26]. The content of thiobarbituric acid reactive substance reflecting the level of lipid peroxidation in tissue homogenate or cell conditioned media was measured with a spectrofluorometer and expressed as MDA amount [27].

### 9. Statistical Analysis

Results were expressed as mean±SEM. The significance of differences between groups was tested with one-way ANOVA followed by the Newman–Keuls test. Linear regression was used to assess the possible correlation between serum ADMA levels and ER stress markers, and Pearson’s correlation coefficients were also calculated. *P*<0.05 was considered as significance.

## Results

### 1. Identification of Type 2 Diabetic Rat Model

**Figure 1:**
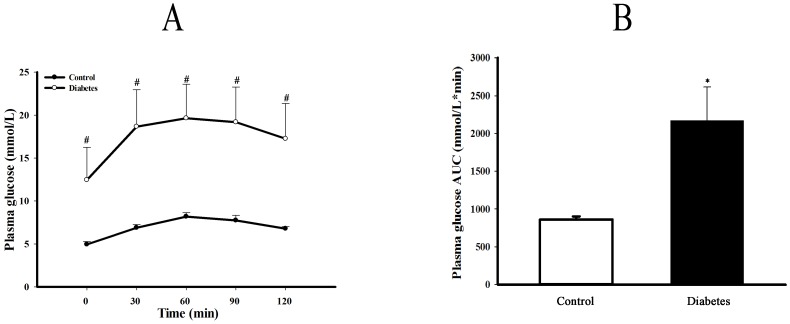
Plasma glucose levels and AUC of OGTT in type 2 diabetic rats. Panel A shows the plasma glucose levels, and panel B is the area under curve (AUC) of plasma glucose concentration during oral glucose tolerance test (OGTT) of control and type 2 diabetic rats. The AUC was calculated by the formula: AUC(mmol/L*min) = 1/2 ×(BG 0 min+BG 30 min)×30 min +1/2×(BG 30 min+BG 60 min)×30 min +1/2×(BG 60 min+BG 90 min)×30 min +1/2×(BG 90 min+BG 120 min)×30 min. Data are expressed as mean ± SEM, n = 5, *P<0.05 *vs* Control.

After 4 weeks high-fat diet feeding plus a low dose streptozotocin (35 mg/kg, i.p) intraperitoneal injection and followed by high-fat feeding for further 8 weeks, type 2 diabetic rat model was identified by OGTT, AUC, FBG, FIns and serum lipid profiles as well as ISI. As shown in figure 1, both FBG and blood glucose concentrations at each time point after oral glucose loading in diabetic rats were significantly higher than that in control rats (*P*<0.05 or 0.01). Resulting AUC of blood glucose concentrations was larger in diabetic group (*P*<0.05). As shown as table 3, either FIns concentration or serum levels of TC, TG and LDL-C except for HDL-C were remarkably elevated in diabetic rats compared to control rats (*P*<0.05 or 0.01). The ISI calculated by both FBG and FIns was decreased (*P*<0.05).

**Table 3:** Changes in blood levels of glucose, insulin, ISI and lipid profiles in diabetic rats.

Groups	Glucose (mmol/L)	Insulin (mmol/L)	ISI (10^−3^)	TG (mmol/L)	TC (mmol/L)	HDL (mmol/L)	LDL (mmol/L)
**Control**	6.89±0.30	10.52±1.10	14.34±1.23	0.41±0.04	1.04±0.12	0.55±0.05	0.22±0.07
**Diabetes**	14.40±2.88*	18.33±2.77*	6.41±1.58**	0.78±0.11*	5.35±1.26**	0.29±0.06*	2.53±0.75*

The levels of fasting blood glucose, plasma insulin and lipid profiles in serum including total cholesterol (TC), triglyceride (TG), low density lipoprotein (LDL) and high density lipoprotein (HDL) were determined in type 2 diabetic rats. Insulin sensitivity index (ISI) was calculated by the formula: ISI = 1/(fasting BG × fasting plasma insulin concentration). Data are expressed as mean ± SEM, n = 5.

*P<0.05,

**P<0.01 *vs* Control.

### 2. ER Stress Provoked in the Liver of Type 2 Diabetic Rats

**Figure 2:**
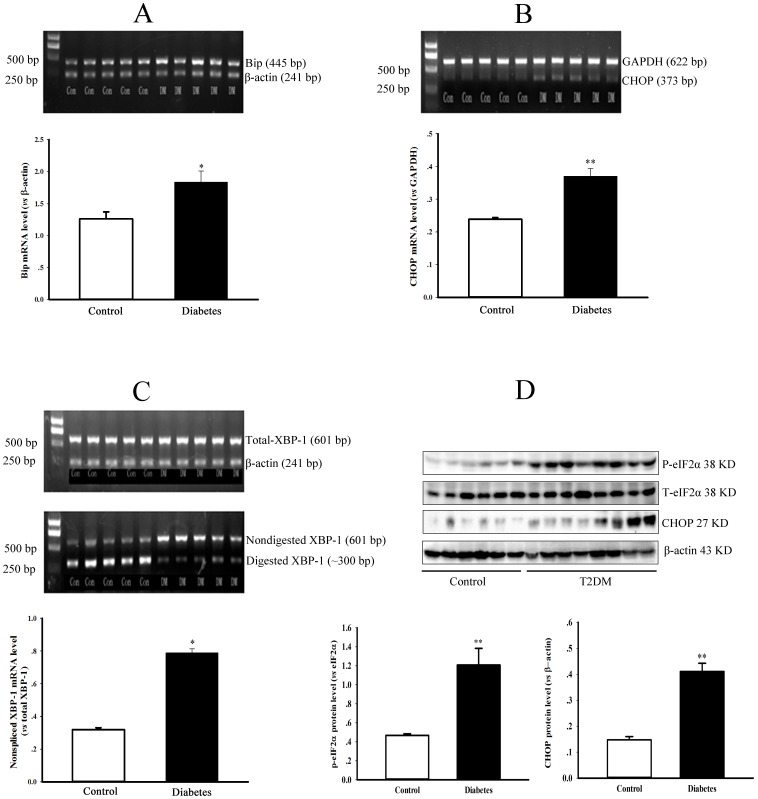
Endoplasmic reticulum stress in the liver of type 2 diabetic rats. Panel A & B show the gel electrophoresis pictures of Bip and CHOP genes PCR products from a representative experiment, and graphs represent the quantification of relative Bip and CHOP mRNA level *vs* internal control gene (β-actin or GAPDH), respectively. Panel C shows the gel electrophoresis pictures of XBP-1 PCR products before (total) and after digesting from a representative experiment. The graph in panel C represents the quantification of relative nonspliced XBP-1 mRNA *vs* total XBP-1 mRNA. Panel D shows the protein expression of phospho-eIF-2α, total-eIF-2α, CHOP and β-actin in the liver of control and diabetic rats detected by Western blotting. Graphs in panel D represent the quantification of relative phospho-eIF-2α or CHOP *vs* total-eIF-2α or β-actin protein levels. Data are expressed as mean ± SEM, n = 5, *P<0.05, **P<0.01 *vs* Control.

It has been widely recognized that the upregulation of Bip, phosphorylation of eIF-2α, splicing of XBP-1 mRNA and expression of CHOP can be used as markers of ER stress. In the present study, the levels of Bip mRNA, eIF-2α phosphorylation, XBP-1 mRNA splicing and CHOP expression were significantly elevated in the liver of type 2 diabetic rats compared to control rats (Figure 2, *P*<0.05 or 0.01).

### 3. The Changes in DDAH/ADMA/NOS/NO Pathway and Redox State in Type 2 Diabetic Rats

**Figure 3:**
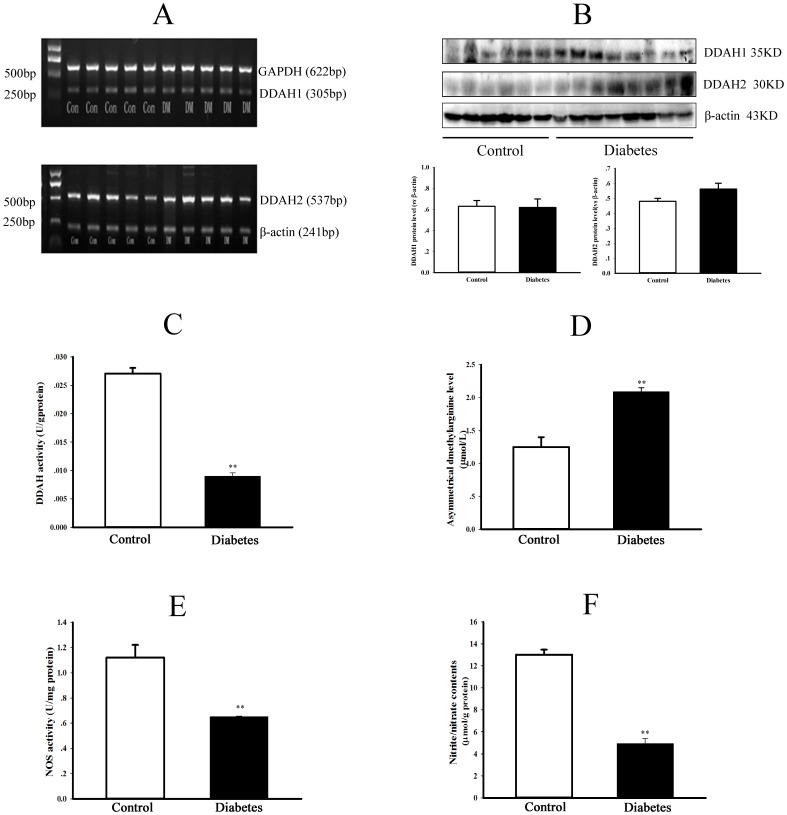
Changes of DDAH/ADMA/NOS/NO pathway in type 2 diabetic rats. Hepatic DDAH1 & DDAH2 mRNA levels (panel A) were detected by RT-PCR, and protein expressions of DDAH1 & DDAH2 (panel B) were detected by Western blotting. The graphs in panel B represent the quantification of relative DDAH1 or DDAH2 *vs* β-actin protein levels. In addition, activities of DDAH (panel C) and NOS (panel E), contents of ADMA in serum (panel D) and nitrite/nitrate in the liver (panel F) were measured in control and diabetic rats. Data are expressed as mean ± *SEM*, n = 5, *P<0.05, **P<0.01 *vs* Control.

As shown in figure 3, although the transcription (Fig 3A) or expression (Fig 3B) of DDAH1 and DDAH2 in liver was not significantly different between diabetic and control group (*P = *NS), the DDAH activity was markedly reduced in the liver of diabetic rats (Fig 3C, *P*<0.01). The serum ADMA level (Fig 3D) was elevated whereas NOS activity (Fig 3E) and NO production reflected by the content of nitrite/nitrate (Fig 3F) were decreased in the liver of diabetic rats (All *P*<0.05).

**Figure 4:**
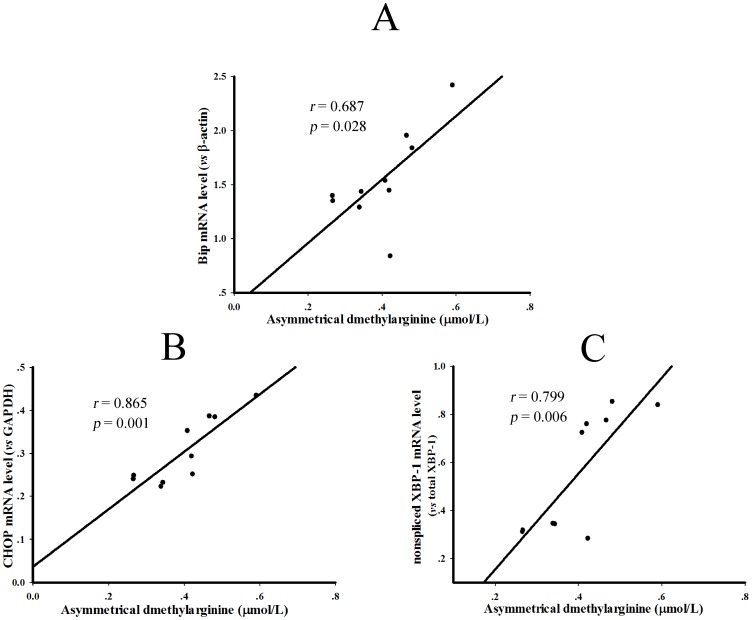
Linear regression analyses between serum ADMA levels and the parameters of hepatic ER stress in the control and diabetic rats. The linear regression analyses were performed between serum ADMA concentrations and transcription of Bip (A), CHOP (B) and the digestion of XBP-1 PCR products (C) in the liver of control and type 2 diabetic rats. The Pearson correlation coefficients were 0.687 (*P* = 0.028), 0.865 (*P* = 0.001) and 0.799 (*P* = 0.006), respectively.

The content of MDA, derived from lipid peroxidation and activity of antioxidant enzyme SOD were detected to reflect the redox state. As shown in figure 4, SOD activity was significantly decreased while MDA content was increased in the liver of diabetic rats as compared with control rats (*P*<0.05).

### 4. The Correlation of Elevated Endogenous ADMA Concentrations and ER Stress in Type 2 Diabetic Rats

**Figure 5:**
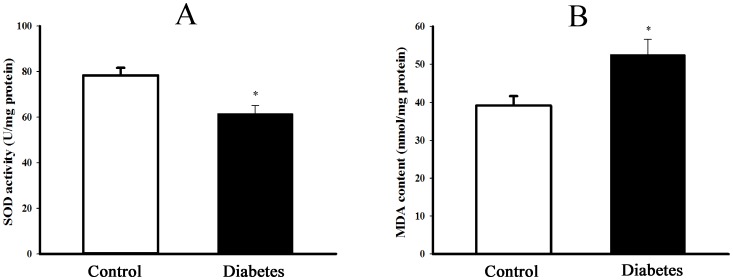
Change of redox state in the liver of type 2 diabetic rats. The content of lipid peroxidation production malondialdehyde (MDA, panel A) and activity of antioxidant enzyme superoxide dismutase (SOD, panel B) were measured to reflect the redox state in the liver of control and type 2 diabetic rats. Data are expressed as mean±*SEM*, n = 5, *P<0.05 *vs* Control.

Linear regression was performed to assess the possible correlation between serum ADMA levels and the parameters reflecting ER stress including Bip mRNA, CHOP mRNA and XBP-1 splicing in type 2 diabetic rats. The results in figure 5A∼C showed that serum ADMA levels and above three indexes were positively correlated, and the Pearson’s correlation coefficients were 0.687, 0.865 and 0.799, respectively (All *P*<0.05).

### 5. Induction of ER Stress in ADMA-treated Hepatocytes

**Figure 6:**
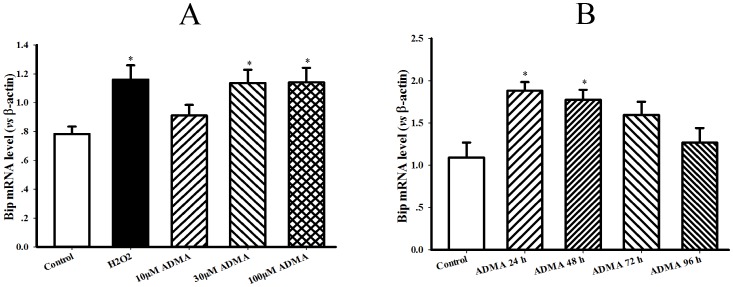
Dose-response and time course of ADMA on Bip mRNA level in hepatocytes. Rat hepatocytes (H4IIE cell line) were incubated with various dose of asymmetric dimethylarginine (ADMA) for different time as indications. Panel A & B present the gel electrophoresis pictures of Bip PCR products from a representative experiment of ADMA dose-response and time course. Graphs in panel A & B represent the quantification of Bip *vs* β-actin from 3 independent experiments, respectively. Date are expressed as mean ± SEM, n = 3, *P<0.05, **P<0.01 *vs* Control.

The concentration-response and time course of ADMA on ER chaperones Bip mRNA level were first investigated in hepatocytes to ascertain the appropriate dose and incubation time of ADMA applied in the following cellular experiments. After respective incubation of H4IIE hepatocytes with 0, 10, 30, 100 µmol/L ADMA for 0, 24, 48, 72, 96 h, Bip transcription was upregulated in a concentration-dependent and time-related manner, especially after exposure to 30 µmol/L ADMA for 48 h (Figure 6A∼B). Accordingly, 30 µmol/L ADMA and 48 h were applied to the following experiments.

**Figure 7:**
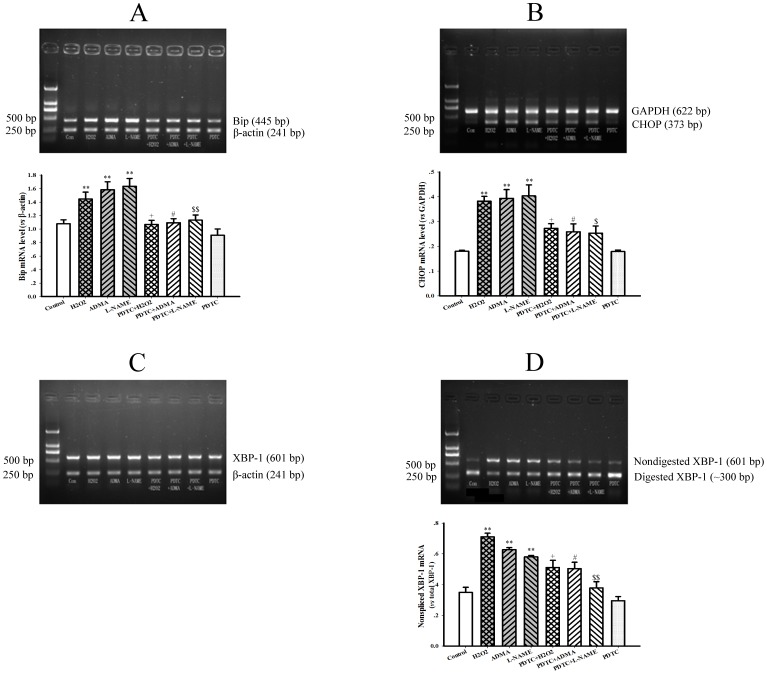
Induction of endoplasmic reticulum stress by ADMA in cultured rat hepatocytes. Rat hepatocytes (H4IIE cell line) were untreated (Control) or treated with 0.5 mM hydrogen peroxide (H_2_O_2_) for 1 h, 30 µM asymmetric dimethylarginine (ADMA), 30 µM N^G^-Nitro-L-arginine Methyl Ester (L-NAME) and 10 µM Pyrrolidine dithiocarbamate (PDTC) for 48 h, or preincubated with 10 µM PDTC for 2 h and then following the co-incubation with 0.5 mM H_2_O_2_ (PDTC+H_2_O_2_) for 1 h, 30 µM ADMA (PDTC+ADMA), 30 µM L-NAME (PDTC+L-NAME) for 48 h. Panel A & B show the gel electrophoresis pictures of Bip and CHOP genes PCR products from a representative experiment, and the graphs in A & B represent the quantification of relative Bip and CHOP mRNA level *vs* internal control gene (β-actin or GAPDH) from 3 independent experiments, respectively. Panel C & D show the gel electrophoresis pictures of XBP-1 PCR products before (total) and after digesting from a representative experiment. The graph in panel D represents the quantification of relative nonspliced XBP-1 mRNA *vs* total XBP-1 mRNA from 3 independent experiments. Data are expressed as mean ± SME, n = 3. **P<0.01 *vs* Control; ^+^ *P*<0.05 *vs* H_2_O_2_; ^#^ *P*<0.05 *vs* ADMA; ^$^ *P*<0.05, ^$$^ *P*<0.01 *vs* L-NAME.

After exposure of hepatocytes to 30 µmol/L ADMA for 48 h, not only the transcription of Bip, but also the splicing of XBP-1 mRNA and the expression of CHOP gene were significantly upregulated (Figure 7 A∼D, all *P*<0.05), indicating that ER stress was provoked in hepatocytes treated with ADMA. Similar results were achieved by incubation of hepatocytes with another exogenous NOS inhibitor L-NAME (30 µmol/L) for 48 h (All *P*<0.05). Moreover, ADMA or L-NAME-induced ER stress in hepatocytes was consistent with the effect of positive control hydrogen peroxide.

### 6. Effects of ADMA on Oxidative Stress, NOS Activity and NO Production in Hepatocytes

**Figure 8:**
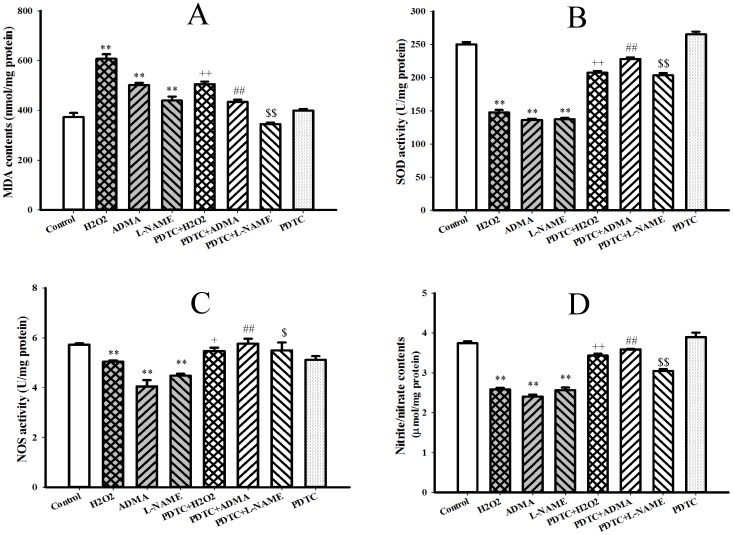
Effects of PDTC on the enhance of oxidative stress and suppression of nitric oxide production in rat hepatocytes induced by ADMA. Rat hepatocytes (H4IIE cell line) were untreated (Control) or treated with 0.5 mM hydrogen peroxide (H_2_O_2_) for 1 h, 30 µM asymmetric dimethylarginine (ADMA), 30 µM N^G^-Nitro-L-arginine Methyl Ester (L-NAME) and 10 µM Pyrrolidine dithiocarbamate (PDTC) for 48 h, or preincubated with 10 µM PDTC for 2 h and then following the co-incubation with 0.5 mM H_2_O_2_ (PDTC+H_2_O_2_) for 1 h, 30 µM ADMA (PDTC+ADMA), 30 µM L-NAME (PDTC+L-NAME) for 48 h. Oxidative stress was reflected by the content of lipid peroxidation production malondialdehyde (MDA, panel A) in the cell conditioned media and the activity of antioxidant enzyme superoxide dismutase (SOD, panel B) in hepatocytes. Nitric oxide (NO) production was evaluated by the activity of nitric oxide synthase (NOS, panel C) in hepatocytes and the content of NO metabolites nitrate/nitrite (panel D) in the cell conditioned media. Data are expressed as mean ± SEM, *P<0.05, **P<0.01 *vs* Control; ^+^ *P*<0.05, ^++^ *P*<0.01 *vs* H2O2; ^##^ *P*<0.01 *vs* ADMA; ^$^ *P*<0.05, *^$$^P*<0.01 *vs* L-NAME.

Incubation of hepatocytes with ADMA also enhanced oxidative stress as reflected by the increase of lipid peroxidation product malondialdehyde contents in condition media (Figure 8A, P<0.01) and the decrease of antioxidant enzyme SOD activities in hepatocytes (Figure 8B, P<0.01). Similar enhancement of oxidative stress was observed after hepatocytes exposure to L-NAME or hydrogen peroxide (Figure 8 A & B, P<0.01). Furthermore, treatment of hepatocytes with ADMA, L-NAME and hydrogen peroxide either suppressed NOS activity (Figure 8 C, P<0.01) or decreased NO production in hepatocytes (Figure 8 D, P<0.01). Pretreatment of hepatocytes with antioxidant PDTC not only attenuated the ER stress induced by hydrogen peroxide, ADMA or L-NAME (Figure 8 A∼D, all *P*<0.05), but also suppressed oxidative stress (Figure 8 A & B, P<0.05) and improved the inhibition of NOS activity and NO production induced by ADMA or L-NAME in H4IIE hepatocytes (Figure 8 C & D, P<0.05). However, PDTC per se neither induced ER stress and oxidative stress nor affected NOS activity and NO production in hepatocytes (Figure 7 & 8, *P* = NS).

## Discussion

In current study, type 2 diabetic rat model was established according to methods described previously [19]. The diabetic rats showed elevated fasting blood glucose and plasma insulin levels, impaired glucose tolerance and reduced insulin sensitivity in accompany with obvious lipid metabolic abnormalities. The phenotype of type 2 diabetic rats in the present study was in consistency with others’ reports previously by similar method [19], [28] and spontaneous type 2 diabetes [29] . These results indicate that type 2 diabetic rat model was successfully established in the present study.

The mechanism underlying type 2 diabetes is not completely understood, but increasing studies had demonstrated that ER stress played an important role in the development of type 2 diabetes [2], [7]–[10], [30]. Diabetic patients are much more susceptible to ER stress than normal individual [31] . In the present study, the increases of Bip transcription, eIF2α phosphorylation, XBP-1 mRNA splicing and CHOP expression was observed in the liver of type 2 diabetic rats. These results indicate that hepatic endoplasmic reticulum stress was provoked in the type 2 diabetic rats of this study. Similar results have been observed in the liver of another type 2 diabetic model db/db mice [32]. Ozcan U et al have also demonstrated that elevated phosphorylation of transmembrane protein IRE1 in the liver of type 2 diabetic model ob/ob mice [33]. Although the phosphorylation of IRE1 was not detected in the present study, its downstream signal, the splicing of XBP-1 mRNA was determined. Since XBP-1 mRNA splicing could facilitate ER-degradation via up-regulating the expression of EDEM, XBP splicing could better reflect ER stress. Altogether, these studies suggest that hepatic endoplasmic reticulum stress plays an important role in the development of type 2 diabetes mellitus.

Previous studies involving ER stress and diabetes were mainly confined to revealing the relationship between them or between ER stress and insulin resistance or β cells apoptosis in various kinds of diabetic animal models. Little is known about the mechanism underlying the induction of endoplasmic reticulum stress in diabetes mellitus. It has been well documented that elevation of endogenous ADMA is prevalent in insulin resistance [16], [17], diabetes [18], [19] and diabetic cardiovascular complication [20]. But whether elevated endogenous ADMA is implicated in the ER stress of diabetes remains unknown. The present study demonstrated for the first time that the elevation of serum ADMA was accompanied by ER stress markers, including the up-regulation of Bip, phosphorylation of eIF2α, splicing of XBP-1 mRNA and expression of CHOP in the liver of type 2 diabetic rats. The linear regression showed a close correlation between the elevated endogenous ADMA and the parameters reflecting hepatic ER stress in type 2 diabetic rats. These results indicate that increased endogenous ADMA is associated with the hepatic ER stress of type 2 diabetic rats.

Since DDAH activity is the key determinant of endogenous ADMA contents, the transcription, expression and activity of DDAH were detected in the present study. It was found that there was no observable difference in the transcription or expression of DDAH1 or DDAH2 in the liver between diabetic rats and normal rats, but the DDAH activity was significantly reduced in diabetic rats, indicating that the accumulation of endogenous ADMA in type 2 diabetic rats was due to the reduction of DDAH activity rather than the suppression of DDAH transcription or expression. Similar results were not only observed in vascular tissue of high-fat diet plus streptozotocin induced type 2 diabetic rats [19], but also found in aorta, liver and kidney of hypercholesteremic rabbits [34]. Therefore, the decreased DDAH activity is the major causes of endogenous ADMA accumulation in metabolic syndrome such as diabetes mellitus and hypercholesterolemia.

To determine the causal relationship between increased endogenous ADMA and hepatic ER stress in diabetes mellitus, rat hepatoma cells (H4IIE) were cultured to investigate direct effects of ADMA on ER stress. Similar ER stress observed in the liver of diabetic rat was discovered in ADMA-treated hepatocytes. These results further indicate that increased endogenous ADMA plays a crucial role in the stimulation of hepatic ER stress in type 2 diabetic rats. Recently, ADMA-induced ER stress also found in cultured lung epithelial cells [35], endothelial cells [36] and 3T3-L1 adipocytes [37]. Furthermore, some well-known factors to elevate endogenous ADMA such as homocysteine [38], free fatty acid [1], [39] and high glucose [40] could induce endoplasmic reticulum stress. Collectively, these studies indicate that ADMA is a trigger for ER stress.

Since oxidative stress is closely related with ER stress [12], [41], and ADMA can induce oxidative stress via NOS uncoupling [21] or activate NF-κB [37], the present study further determine whether oxidative stress is implicated in the ADMA-induced ER stress in cultured hepatocytes. It was found that both ADMA and another exogenous NOS inhibitor L-NAME not only induced ER stress but also caused oxidative stress as assessed by the increase of malondialdehyde content in conditional media and the decrease of SOD activity in hepatocytes. Their actions were very consistent with the effects of positive control hydrogen peroxide, which has been proven to cause ER stress in a variety of cells [42]. Most importantly, both ER stress and oxidative stress induced by ADMA, L-NAME or hydrogen peroxide could be significantly attenuated by the treatment of PDTC, which has been generally considered as an antioxidant and inhibitor of NF-κB activation. PDTC is a thiol-containing compound with the strong ability of capturing unpaired electrons and could directly scavenge oxygen free radical or increasing the contents of reduced glutathione [43]. It has been demonstrated that PDTC could inhibit interferon-γ-induced ER stress in primary cultured murine hepatocytes [13]. Another antioxidant N-acetylcysteine, without NF-κB inhibition, has also reported to inhibit arsenite-induced ER stress in dorsal root ganglion explants [14] and cadmium-induced ER stress in germ cells of testes [15]. Taken together, these results suggest that the increase of oxidative stress may be involved in ADMA-induced ER stress in hepatocytes. In addition to an increase of oxidative stress, this study also presented a decrease of NO production reflected by the inhibition of NOS activity and reduction of NO metabolic product nitrite/nitrate content in both ADMA-treated hepatocytes and the liver of type 2 diabetic rats. Therefore, it would be speculated that ADMA-induced oxidative stress might be due to the NOS uncoupling, which could uncouple the electron transfer between L-arginine and NOS via NOS inhibition resulting in the increases of superoxide production [21].

There are also some limitations in the present study, such as *in vivo* experiments only observed the association between elevated endogenous ADMA and hepatic ER stress in type 2 diabetic rats and did not use any intervention, even though the cell culture experiments show the direct effect of ADMA on ER stress in hepatocytes. If the liver-specific overexpression of an ADMA-lowering enzyme DDAH had performed in type 2 diabetic rats, it would have made this study perfect. In addition, it has been recently demonstrated that ADMA can be metabolized in liver via the transamination by the enzyme alanine-glyoxylate aminotransferase 2 (AGXT2) [44]. This study did not measure the activity of AGXT2 to assess its role in endogenous ADMA accumulation in T2DM. Although DDAH is generally considered as the major enzyme responsible for more than 80% of ADMA hydrolysis *or elimination,* only less than 20% of ADMA metabolism counts on renal excretion and AGXT2, which is thought as a mitochondrial aminotransferase expressed primarily in the kidney [44], if the activities of both enzymes had been measured in the liver of T2DM rats, it would have made this study more perfect. Furthermore, the *in vitro* experiments did not use ADMA analogue symmetric dimethylarginine (SDMA) as control in culture hepatocytes. Even though generally consider that SDMA does not have many roles of ADMA and most studies on ADMA were rarely used SDMA as control, if authors had added SDMA into the cell culture experiments as the chemical control, it would have made the present study more rigorous and also illustrated that the produced phenotypes are indeed ADMA-specific in culture hepatocytes.

In summary, the present study first reveals that elevated endogenous ADMA contributes to hepatic ER stress in type 2 diabetic rats and then discover that increased ADMA is the direct cause of ER stress in cultured rat hepatocytes. Treatment with antioxidant PDTC not only suppressed ADMA-induced ER stress, but also attenuated ADMA-induced oxidative stress in combining with the inhibition of NO production, indicating that the mechanism underlying ADMA-induced ER stress may relate to oxidative stress via NOS uncoupling. These results from the present study provide the new insight into the mechanism for ER stress provoked in diabetes mellitus and help us to better understand the pathophysiological roles of ADMA.
